# Non-Invasive Methods to Monitor Mechanisms of Resistance to Tyrosine Kinase Inhibitors in Non-Small-Cell Lung Cancer: Where Do We Stand?

**DOI:** 10.3390/ijms17071186

**Published:** 2016-07-22

**Authors:** Paola Ulivi

**Affiliations:** Biosciences Laboratory, Istituto Scientifico Romagnolo per lo Studio e la Cura dei Tumori (IRST) IRCCS, Meldola 47014, Italy; paola.ulivi@irst.emr.it; Tel.: +39-0543-739277

**Keywords:** lung cancer, TKI-resistance, liquid biopsy, EGFR, EML4–ALK

## Abstract

The induction of resistance mechanisms represents an important problem for the targeted therapy of patients with non-small-cell lung cancer (NSCLC). The best-known resistance mechanism induced during treatment with epidermal growth factor receptor (EGFR)-tyrosine kinase inhibitors (TKIs) is *EGFR* T790M mutation for which specific drugs are have been developed. However, other molecular alterations have also been reported as induced resistance mechanisms to EGFR-TKIs. Similarly, there is growing evidence of acquired resistance mechanisms to anaplastic lymphoma kinase (ALK)-TKI treatment. A better understanding of these acquired resistance mechanisms is essential in clinical practice as patients could be treated with specific drugs that are active against the induced alterations. The use of free circulating tumor nucleic acids or circulating tumor cells (CTCs) enables resistance mechanisms to be characterized in a non-invasive manner and reduces the need for tumor re-biopsy. This review discusses the main resistance mechanisms to TKIs and provides a comprehensive overview of innovative strategies to evaluate known resistance mechanisms in free circulating nucleic acids or CTCs and potential future orientations for these non-invasive approaches.

## 1. Introduction

Tyrosine kinase inhibitors (TKIs) represent a group of anticancer agents showing important results in the treatment of patients with non-small-cell lung cancer (NSCLC) harboring specific molecular alterations. In particular, epidermal growth factor receptor (EGFR)-TKIs are capable of improving response and prolonging progression-free survival (PFS) in selected patients harboring specific *EGFR* mutations occurring principally at exons 18, 19 and 21 [1,2,3,4]. Gefitinib [2], erlotinib [3] and, more recently, afatinib [5], are the only three TKIs approved for the first-line treatment of *EGFR*-mutated advanced NSCLC, which represents about 10%–15% of all NSCLC. Alterations of the anaplastic lymphoma kinase (ALK), present in about 5% of NSCLC, define another subgroup of tumors that are highly responsive to ALK-TKIs, such as crizotinib [6]. The objective response rate (ORR) of these drugs in selected patients is about 75%–80% and randomized clinical trials have also reported improved PFS [1,2,3,6]. However, the long-term effectiveness of these therapies is usually limited by the development of resistance mechanisms that consequently lead to disease progression within one year of starting treatment [1,2,3,7,8]. The characterization and identification of such mechanisms is an important field of research and new drugs capable of overcoming resistance are beginning to emerge. Furthermore, as recurrent NSCLC lesions can only be characterized by performing bronchoscopy, the development of non invasive strategies for this purpose would constitute an important step forward in this setting. Free circulating tumor nucleic acids or circulating tumor cells (CTCs) represent one such strategy.

## 2. Acquired Resistance Mechanisms to Epidermal Growth Factor Receptor (EGFR)-Tyrosine Kinase Inhibitors (TKIs) and Possible Strategies for Their Inhibition

### 2.1. Gatekeeper EGFR T790M Mutation

The mechanisms of resistance induced by EGFR-TKI treatment in *EGFR*-mutated NSCLC patients have been widely investigated. The main molecular alteration induced in over 50% of patients who become resistant to TKI is the threonine-to-methionine substitution within the gatekeeper residue at amino acid position 790 (T790M) of the *EGFR* gene [9]. Threonine 790 has been designated as a “gatekeeper” residue and is important for regulating inhibitor specificity in the adenosine triphosphate (ATP) binding pocket. The T790M mutation enhances affinity of the ATP binding pocket for ATP, thus successfully competing with TKIs and ultimately conferring resistance. Tumors carrying *EGFR* mutation are usually sensitive to competitive inhibitors as such mutations reduce the receptor’s affinity for ATP. The onset of T790M re-established the ATP affinity of the kinase back to wild-type levels, restoring ATP as the favored substrate instead of the TKI [10]. Tumors developing this alteration are usually more indolent [11] and patients tend to have longer post-progression survival (PPS) than those without the mutation [12]. Given that tumor cells harboring a T790M mutation are still addicted to the EGFR signaling pathway, new drugs that irreversibly block EGFR, e.g., second-generation TKIs, may be capable of increasing the potency of EGFR-TK inhibition. One such inhibitor, the second-generation EGFR-TKI afatinib (BIBW-2992), is capable of selectively blocking both wild-type and mutant forms of ErbB family receptors (EGFR, ErbB2, ErbB3 and ErbB4) [10]. However, despite initially promising results reported in some clinical studies [13,14], the potential of afatinib appears to be somewhat weakened due to toxicity and insufficient blood concentrations that fail to overcome the *EGFR* T790M mutation [15]. Thus, several third-generation EGFR-TKIs selectively targeting the mutant *EGFR* (in particular, the T790M mutation) but with minimal potency towards the wild-type receptor have emerged in quick succession [16,17]. The pyrimidine compound AZD9291, a potent, irreversible EGFR inhibitor that targets via covalent binding the cysteine-797 residue in the ATP binding site [17,18], has showed strong activity in different in vitro models carrying *EGFR* mutation with or without T790M [17]. This agent was studied in a phase I trial in patients with *EGFR*-mutant advanced NSCLC who progressed during EGFR-TKIs treatment. Median PFS in patients with or without a confirmed T790M was 9.6 months (95% confidence interval (CI), 8.3 months–not reached) and 2.8 months (95% CI, 2.1–4.3 months), respectively [19]. Given the proven activity and favorable toxicity profile of AZD9291 in patients with *EGFR*-mutant NSCLC who progress on EGFR-TKIs, approval by regulatory agencies is expected in the near future. However, other resistance mechanisms to AZD9291 have also been reported [20].

### 2.2. Mesenchimal–Epithelial Transition (MET) Amplification

The mesenchymal–epithelial transition (MET) receptor, a transmembrane tyrosine kinase encoded by the proto-oncogene *MET*, has been acknowledged as one of the main causes of acquired resistance to gefitinib or erlotinib in NSCLC. Once bound to its ligand (hepatocyte growth factor), MET tyrosine kinase phosphorylation is induced, triggering the activation of the downstream PI3K/AKT/mTOR pathway, one of the key signaling pathways for cell proliferation, survival and anti-apoptosis [21]. About 20% of tumors with EGFR-TKI acquired resistance have been shown to possess *MET* gene amplification [21,22] and some strategies have been studied to inhibit MET activity. Tivantinib is a non-ATP-competitive small molecule MET inhibitor that showed promising results in the MARQUEE trial [23]. However, the efficacy of the drug would not seem to be related to MET expression [24]. Other strategies include the use of monoclonal anti-MET antibodies. Onartuzumab (MetMAb), a newly developed humanized monoclonal antibody targeting MET, prevents hepatocyte growth factor from binding to MET, inhibiting the activation of its downstream transducers and effectors [25]. However, a recent phase III trial failed to show any benefit from the drug plus erlotinib compared to erlotinib only in MET-positive patients [26].

### 2.3. Insulin-Like Growth Factor-1 Receptor (IGF-1R)

Higher insulin-like growth factor-1 receptor (IGF-1R) expression levels have been detected in patients with acquired gefitinib resistance than in those who are sensitive to the drug [27]. The mechanisms through which IGF-1R is activated are still unknown. The activation of this receptor induces survival signals such as PI3K/AKT and MAPK to activate the mammalian target of rapamycin (mTOR), inducing the synthesis of EGFR and anti-apoptotic survivin proteins [28]. The concomitant treatment of IGF-1R inhibitors such as α-IR3, AG1024 or R1507 with EGFR-TKIs may enhance TKI-induced growth inhibition and apoptosis, representing a potential strategy for overcoming primary resistance to EGFR-TKIs in NSCLC [29,30].

### 2.4. Human Epidermal Growth Factor Receptor (HER) 2 Mutations

*Human Epidermal Growth Factor Receptor 2* (*HER2*) mutations are present in about 2% of lung adenocarcinomas and induce the constitutive activation of HER2 protein, leading to the activation of EGFR through heterodimerization [31]. This mechanism may lead to EGFR activation, independently of the action of EGFR-TKIs [31,32]. Moreover, EGFR-TKI resistance can be mediated by *HER2* gene amplification or protein overexpression [33], providing a rationale for the use of HER2-targeted agents such as lapatinib, trastuzumab or dacomitinib in combination with EGFR-TKIs to treat NSCLC patients who develop *HER2* alterations [34].

### 2.5. Activation of Alternative Proliferation Pathways

The PI3K/AKT/mTOR signaling pathway, which is downstream to the activation of tyrosine kinase receptors (EGFR, MET, HER2, HER3), plays an important role in promoting the proliferation, survival and drug resistance of cancer cells [35]. Some studies have also reported that the involvement of mTOR pathway in specific diseases causes defects in translation and that stimulation of this pathway would seem to have therapeutic potential [36,37,38]. Although there is little clinical data on the prevalence of PI3K/AKT/mTOR pathway-induced EGFR-TKI resistance, the involvement of this pathway in induced EGFR-TKI resistance has been repeatedly confirmed by preclinical research [39,40]. However, *PIK3CA* mutations have been identified in a small percentage of EGFR-mutant lung cancers acquiring resistance to EGFR-TKIs [41]. Although the combination of EGFR-TKIs and PIK3CA inhibitors is a potential challenge because of overlapping toxicities, it is currently being evaluated in a number of clinical trials [42].

### 2.6. Phenotypic Changes

The phenotypic transformation of NSCLC histology into small cell lung cancer (SCLC) was demonstrated for the first time by Sequist et al. [41] who used immunohistochemical staining for synaptophysin in post-resistance biopsy specimens to confirm positivity for the SCLC phenotype [41]. Molecular analysis also showed that the *EGFR* mutation was maintained in the newly-developed SCLCs. It can be hypothesized that SCLC cells originate from minor pre-existing cells under the selective pressure of EGFR-TKIs, transdifferentiate from adenocarcinoma cells, or arise from multipotent stem cells [43].

Another documented phenotypic change responsible for acquired resistance to TKIs is the epithelial to mesenchymal transition (EMT). Typical molecular events characterizing this phenomenon are the loss of epithelial cell junction proteins such as E-cadherin and the acquisition of mesenchymal markers such as vimentin or fibronectin [44]. It has been demonstrated that EMT accounts for about 5% of all cases of EGFR-TKI resistance [41]. However, NSCLC cells with a lower degree of EMT but no *EGFR*-mutations have also shown sensitivity to EGFR-TKIs in both in vitro and xenograft models [45,46].

## 3. Acquired Resistance Mechanisms to Anaplastic Lymphoma Kinase (ALK)-TKIs

### 3.1. ALK-Dependent Mechanisms

#### 3.1.1. Secondary Mutations at the *ALK* Gene

The first major “gatekeeper” mutation identified in the TK domain of echinoderm microtubule associated protein like 4 (EML4)-ALK was described in an ALK-positive patient who progressed after 5 months’ treatment with crizotinib. Analysis of the patient’s pleural fluid revealed two non-overlapping mutations in the ALK kinase domain (L1196M and C1156Y), each independently conferring crizotinib resistance in vitro [47]. The substitution of leucine for a methionine at position 1196 (L1196M) induced a mutant bulky amino-acid side chain in the ATP-binding pocket of the receptor, interfering with the binding of crizotinib to its receptor. Subsequent analyses performed on tumor tissue acquiring resistance to crizotinib revealed other “non-gatekeeper” second-site ALK mutations distributed throughout the kinase domain [48,49,50]. These gatekeeper mutations inhibit binding of crizotinib to the kinase domain of the ALK receptor, leading to its ineffectiveness. However, not all secondary *ALK* mutations have the same impact on the efficacy of anti-ALK agents. The presence of G1202R *ALK* mutation confers high-level resistance not only to crizotinib but also to next-generation ALK inhibitors, whereas the L1196M mutation may still be sensitive to newer ALK inhibitors such as CH5424802 [51].

#### 3.1.2. ALK Amplification

An increase in *ALK* gene fusion copy number was recently implicated as a cause of crizotinib resistance. This phenomenon was initially observed in cell line models in which the amplification of wild-type EML4–ALK was sufficient to induce crizotinib resistance, and later confirmed in resistant clinical biopsy specimens [52,53].

### 3.2. ALK-Independent Mechanisms

The activation of ALK-independent signaling pathways contributes to acquired resistance to ALK-TKIs in up to 20% of ALK-positive NSCLCs [54]. These mechanisms include activation of EGFR, HSP90, PI3K/AKT/mTOR pathways, V-Ki-ras2 Kirsten rat sarcoma viral oncogene homolog (*KRAS*) mutations and *KIT* amplifications. With regard to EGFR, increased phosphorylation of the receptor and upregulation of EGFR ligands, e.g., EGF and amphiregulin, have been observed [55,56]. It has also been hypothesized that activation of autophagy through the AKT/mTOR signaling pathway may contribute to drug resistance and that the inhibition of autophagy with chloroquin may restore sensitivity to crizotinib in ALK-resistant cell lines [57]. Finally, *KRAS* mutations and *KIT* gene amplification have been identified in ALK-positive patients during treatment with crizotinib, suggesting that these signaling pathways may also contribute to the develop of resistance [50].

## 4. Use of Non-Invasive Methods for the Detection of Resistance Mechanisms to TKIs

**Figure 1:**
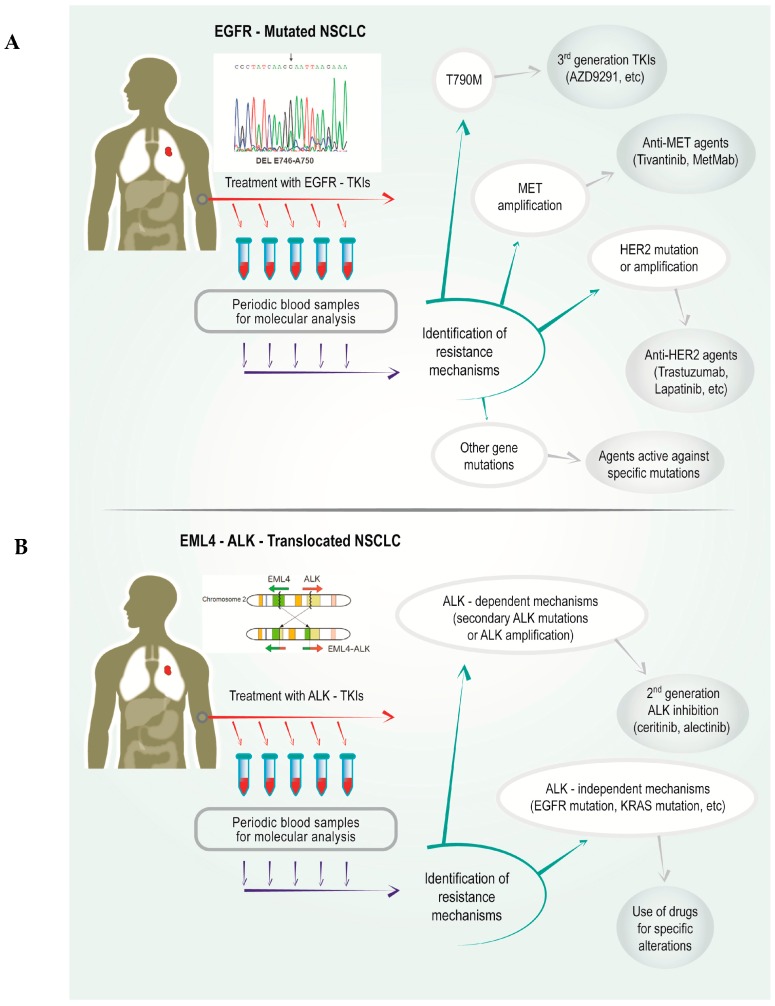
Schematic representation of the possible impact of liquid biopsy analysis during the course of treatment in (**A**) an epidermal growth factor receptor (*EGFR*)-mutated patient treated with an EGFR- tyrosine kinase inhibitors (TKIs) and (**B**) a patient with echinoderm microtubule associated protein like 4–anaplastic lymphoma kinase (*EML4–ALK*) translocation treated with an ALK-TKI. NSCLC, non-small-cell lung cancer; MET, mesenchymal–epithelial transition; HER2, human epidermal growth factor receptor 2.

The identification of molecular alterations responsible for acquired TKI resistance is important from a clinical point of view as subsequent targeted treatment can then be given (Figure 1). At present, such alterations can only be characterized by biopsy of the recurrent lesion, an invasive procedure that is not without risk for patients. Moreover, as tumors are heterogeneous, a single biopsy sample taken from a specific area of the lesion may not be fully representative of the tumor as mutations may be present in some areas but not in others. The selective pressure induced by treatment with EGFR-TKI can also contribute to increasing heterogeneity. An alternative could be to identify the specific alteration by non-invasive methodologies, e.g., free circulating tumor DNA (fctDNA) or CTCs. Although these non-invasive approaches may not be as sensitive as tumor tissue analysis, they nevertheless offer important advantages. First, fctDNA and CTCs may be more representative of the overall tumor area as they can be released from any part of the tumor mass, making it easier to identify mutations present. These methods also enable patients to be monitored during treatment, leading to the timely identification of the onset of treatment resistance [58]. Numerous studies have been performed to verify the potential of non-invasive strategies to identify induced resistance mechanisms to TKIs.

### 4.1. EGFR Mutations

A great deal of research has been performed to identify *EGFR* T790M mutations and to monitor EGFR-sensitive mutations in fctDNA or CTCs during the course of TKI treatment [59,60,61,62,63,64,65,66,67,68,69] (Table 1).

A recently published study used droplet digital PCR (ddPCR) to analyze plasma from 79 NSCLC patients treated with TKIs and monitored every 8 weeks until progression [59]. The clinical sensitivity of *EGFR* mutation detection in plasma, assessed in 58 patients for whom baseline samples were available, was 74%, with 100% specificity. Baseline *EGFR* mutation positivity in 40 patients decreased to an undetectable mutation level during treatment. The authors also found that PFS and overall survival (OS) were longer in patients with undetectable *EGFR* mutations (10.1 and 23.7 months, respectively) than in those with detectable mutations (6.3 and 11.2 months, *p* = 0.006 and *p* = 0.001, respectively). Among the 49 patients with sample availability at progression, 14 (28.6%) had T790M mutations, 8 of which were detected 2–12 months prior to radiological progression and 6 at the time of progression [59]. Another recent study reported that 58/87 *EGFR*-mutated patients treated with a TKI progressed, 23 (40%) showing T790M mutations in plasma. The mutations were detected over a period ranging from 11 months pre-progression to 9 months post-progression [60]. T790M mutation was more frequent in males, smokers and patients with exon 19 deletion. Moreover, T790M-negative patients who progressed showed a longer OS than T790M-positive patients (median 782 vs. 516 days, respectively) [60]. In contrast, another study performed on 23 patients reported T790M mutations in 39% of cases. The T790M-positive group showed a time-to-progression of 341 days compared to 152 days for those without T790M alterations. In the same study, T790M mutation was detectable up to 344 days before clinically evident disease progression [62]. In another study, an increase in T790M alteration during TKI treatment was associated with a longer PFS and OS than a decrease in the mutation rate [64].

Other recent results obtained on 117 *EGFR*-mutated NSCLC patients acquiring resistance to TKIs revealed that T790M was detectable in 47% of patients, almost half of whom showed the mutation a median of 2.2 months before clinically evident progressive disease. Moreover, T790M-positive patients had a significantly shorter OS (26.9 months) then those with no T790M mutation (not reached, *p* = 0.05) [65]. However, another study did not report any difference in PFS between T790M-positive and -negative patients [66].

Overall, conflicting results emerge from the studies published on the prognostic role of T790M mutations, some correlating them with a poorer prognosis [60,65], some with a more favorable prognosis [63], and others reporting no difference in survival between T790M-positive and -negative patients [66]. However, studies on tissue re-biopsy would seem to confirm that patients acquiring the T790M mutation have a better outcome than those who do not develop this resistance mechanism [11,12]. Moreover, results from an in vitro study showed that T790M-positive cell lines had different growth kinetics than those without the mutation, i.e., a slower proliferation rate and consequently more indolent tumor growth [70].

Recently, Sundaresan et al. compared tumor biopsy, fctDNA and CTCs for their potential to identify T790M mutations [67], reporting detection rates of 47%, 50% and 50%, respectively. The authors concluded that, although the combined use of fctDNA and CTCs could effectively increase the rate of T790M identification, the high cost of CTC analysis represents a major limitation to its use in clinical practice.

Other authors have shown that monitoring *EGFR* mutations may also be useful in patients who develop the T790M mutation during treatment with third-generation TKIs [71,72]. In particular, Thress et al. reported that fctDNA analysis during treatment with AZD9291 permitted tumor molecular characterization and led to the identification of *EGFR* C797S mutation as an acquired resistance mechanism to the drug [72].

Results from other studies underline the possibility of quantifying *EGFR*-sensitizing mutations, even after only a few days’ treatment, to predict response to TKIs [73,74,75]. In the study by Tseng et al. on 62 *EGFR*-mutant patients treated with TKIs, failure to clear plasma *EGFR* mutations (evaluated by peptide nucleic acid-zip nucleic acid polymerase chain reaction clamp method) after treatment was found to be an independent predictor of lower disease control rate and shorter PFS and OS [74]. More specifically, Marchetti et al. evaluated 69 *EGFR*-mutated patients using next generation sequencing (NGS) and PCR-based approaches, observing that a decrease in plasma *EGFR* mutation during the first few days of treatment was closely correlated with response. In particular, the authors found that mutations began to decrease from the fourth day of treatment onwards and showed an average decrease of 63.5% after 14 days. In rapid responders, a rapid decrease in mutated plasma *EGFR* DNA was associated with high levels of tumor reduction after a few months, and no T790M mutation was observed in plasma. Conversely, slow responders showed a high frequency of T790M mutations, the authors hypothesizing a greater susceptibility of this group to develop early resistance [73].

### 4.2. MET Amplification

Relatively few studies have been performed to analyze *MET* amplification during treatment with TKIs [62,64]. In the study by Wang et al., *MET* amplification was detected in 19% of patients who progressed during TKIs and developed a T790M mutation. The percentage of *MET* amplification was higher (32%) in patients who showed a decrease in T790M during treatment than in those with an increase in the mutation (12%) [64]. Conversely, Ishii et al. did not observe *MET* gene copy number gain in plasma of 18 *EGFR*-mutated patients with acquired resistance to TKIs [62].

### 4.3. KRAS Mutations

*KRAS* mutations would not seem to be involved in creating primary resistance to TKIs in NSCLC [76,77,78]. However, a recent study evaluating *KRAS* in fctDNA of 33 *EGFR*-mutated patients receiving TKIs found that 16 (49%) had a plasma *KRAS* mutation at codon 12 at progression. Moreover, 12/16 patients had a concomitant *EGFR* T790M mutation. Further research is warranted to clarify the role of KRAS in this context [79,80].

### 4.4. ALK Alterations

There is increasing evidence of the potential for detecting ALK rearrangements using non-invasive methods [81,82,83,84,85]. The first study focusing on the identification of *EML4–ALK* translocation was performed by Ilie et al. who used a method for CTC isolation based on size (isolation by size of epithelial tumor cells, ISET) and then evaluated ALK by fluorescent in situ hybridization (FISH) and immunohistochemistry (IHC) methods [81]. All five patients with ALK-positive tumors showed both IHC and FISH ALK positivity in CTCs, demonstrating 100% sensitivity, and no ALK-positive CTCs were found in patients with ALK-negative tumors. Subsequently, Pailler et al. [82] aimed to identify the translocation in CTCs of patients with EML4–ALK-positive NSCLC using a filtration enrichment technique and filter-adapted fluorescent in situ hybridization (FA-FISH) method. All 18 ALK-positive patients tested were positive for ALK-rearranged CTCs, with 100% sensitivity and nearly absolute specificity. Conversely, using the CellSearch methodology, CTCs were found in only 6 (35%) of the 17 samples analyzed. The authors also found that ALK-rearranged CTCs had a homogeneous mesenchymal phenotype, independently of the heterogeneous marker expression observed in the tumor specimens. Moreover, 5 patients receiving crizotinib were monitored for ALK-rearranged CTCs, the authors confirming that quantitative and qualitative analysis of circulating tumor cells was feasible during treatment [82].

Two other recent reports suggested that CTC analysis may be useful in detecting EML4–ALK rearrangement [83,84]. He et al. demonstrated the superiority of the CTC-enrichment method NanoVelcro over the CellSearch methodology, reporting 100% sensitivity of the former in detecting EML4–ALK translocation in 21 ALK-positive patients analyzed. The authors also confirmed that it was possible to monitor patients during the course of treatment [83]. Similarly, another study using the ClearCell FX system to isolate CTCs reported a high concordance of the method in detecting ALK-rearranged CTCs in ALK-positive patients. Patient monitoring during treatment revealed ALK FISH rearrangement suggestive of gene copy number increase in those who progressed [84].

Another recent study by Nilsson et al. showed that the detection of *EML4–ALK* translocation is feasible in RNA isolated from circulating blood platelets [85]. The authors reported that plasma RNA translocation was only evaluable in 3 out of 14 *EML4–ALK* rearranged tumors, indicating a sensitivity of 21% and a specificity of 100%. Conversely, the evaluation of platelet-derived RNA in 34 patients with *EML4–ALK* translocation showed a sensitivity of 65% and maintained 100% specificity. Moreover, the authors found that the presence of *EML4–ALK* translocation in platelets was associated with shorter PFS and OS. No differences in terms of response to crizotinib were observed between patients with circulating *EML4–ALK* positivity or negativity. One patient in whom the translocation was detected in platelets was monitored, the authors reporting that the alteration disappeared during treatment but re-appeared 2 months before radiological progression was documented [85]. This is suggestive of the development of ALK-independent resistance mechanisms.

The identification of *EML4–ALK* translocation by non-invasive methods seems feasible, with CTCs possibly the best option as morphological characteristics of cells can also be evaluated, providing information that can be correlated with response to therapy. Moreover, CTC analysis appears to be more accurate for translocation detection as it shows absolute sensitivity and specificity and methods such as filter-adapted fluorescent in situ hybridization (FA FISH), NanoVelcro or ClearCell FX would seem to be more efficient than CellSearch. However, further studies are needed to identify the best methods for detecting circulating *EML4–ALK* and to verify whether the identification of the translocation is useful in monitoring response to crizotinib.

The analysis of *ALK* alterations in liquid biopsy could also be integrated by the search for *ALK* mutations recognized as mechanisms of resistance to crizotinib in tumor tissue, e.g., L1196M and C1156Y [47], or for other known mutations distributed throughout the kinase domain [48,49,50]. The mutations in question are somatic mutations and could thus be evaluated in fctDNA using the same methodologies as those employed for the detection of *EGFR* T790M alterations. The identification of resistance mechanisms induced during crizotinib treatment would help us to understand whether the tumor has developed ALK-dependent or -independent resistance and could orient the choice of subsequent treatment accordingly.

## 5. Conclusions and Future Directions

The analysis of *EGFR* mutations in fctDNA represents a validated methodology that is now ready for use in clinical practice to identify resistance to TKIs (by T790M analysis) or to monitor TKI response after only a few days’ treatment (through the detection of *EGFR*-sensitizing mutations). The fact of being able to identify somatic point mutations in fctDNA means that all the mutations responsible for creating resistance can be detected and monitored in a non-invasive way. As fctDNA evaluation is a relatively simple, inexpensive and rapid approach, it could easily be implemented in clinical practice. In fact, all the DNA mutations with a proven role in causing TKI resistance can be determined in fctDNA. However, as there are still no drugs available for each single alteration causing TKI resistance, the usefulness of fctDNA in clinical practice is, for the moment, somewhat limited. The analysis of other types of alterations not identifiable in DNA that require RNA isolation is slightly more problematic as free RNA is not as stable as DNA. However, the fact that some studies have shown the feasibility of analyzing *EML4–ALK* translocation in CTCs [81,82,83,84] or in RNA isolated from circulating blood platelets [85] has opened up interesting new avenues of research. The same non-invasive approaches could also potentially be used to search for c-ros oncogene 1 (ROS1) rearrangements. Pending the development of new drugs targeting mutations responsible for resistance, further research is warranted into the evaluation of the prognostic role of fctDNA alterations as potential indicators of TKI response and patient outcome.

In conclusion, despite the acknowledged advantages of using liquid biopsy (it is a non-invasive approach, representative of the heterogeneous nature of the tumor, and can be used to monitor patients during treatment) which facilitate the timely detection of the onset of resistance mechanisms, further efforts are needed to increase the sensitivity of the methodologies to reduce the risk of false-negative results.

## Figures and Tables

**Table 1:** Literature data on T790M identification in free circulating tumor DNA (fctDNA) during tyrosine kinase inhibitors (TKI) treatment.

Author (Reference)	Year	Methodology	No. of Patients *	T790M Detected in Plasma (%)	Concordance between T790M in Plasma/CTCs and Tumor Re-Biopsy (%)	T790M Identified Prior to Clinical Disease Progression
Lee et al. [59]	2016	ddPCR	79	29	NE	Yes
Sueoka-Aragane et al. [60]	2016	MBP-QP	87	40	50	Yes
Seki et al. [61]	2016	Picoliter ddPCR	35	44	80	NE
Ishii et al. [62]	2015	ddPCR	18	56	83	NE
Sorensen et al. [63]	2014	Cobas EGFR blood test	23	39	NE	Yes
Wang et al. [64]	2014	DHPLC	135	43	NE	NE
Zheng et al. [65]	2016	ddPCR	117	47	NE	Yes
Sakai et al. [66]	2013	SABER-Sequenom MassARRAY	75	28	NE	NE
Sunderasan et al. [67]	2016	CTC-enriched PCR method	28	50	57/74 ^†^	NE
		fctDNA-Cobas EGFR mut test	32	50	60/61 ^†^	NE
		CTCs and fctDNA together	23	100	65/69 ^†^	NE
Marcq et al. [68]	2014	ARMS	2	50	NE	NE
Del Re et al. [69]	2016	ddPCR	33	33	62.5	NE

* Number of epidermal growth factor receptor (EGFR)-mutated patients treated with TKIs and monitored for T790M mutation; ^†^ percentages refer to multiple re-biopsies; NE, not evaluable; CTCs, circulating tumor cells; ddPCR, droplet digital PCR; MBP-QP, mutation-biased polymerase chain reaction (PCR) quenching probe; DHPLC, denaturing high performance liquid chromatography; ARMS, amplification refractory mutation system.
